# Recent advances in understanding acute respiratory distress syndrome

**DOI:** 10.12688/f1000research.11148.1

**Published:** 2018-03-05

**Authors:** Peter Wohlrab, Felix Kraft, Verena Tretter, Roman Ullrich, Klaus Markstaller, Klaus Ulrich Klein

**Affiliations:** 1 Department of Anaesthesia, General Intensive Care and Pain Management, Medical University of Vienna, Währinger Gürtel 18-20, 1090 Vienna, Austria

**Keywords:** acute respiratory distress syndrome, ventilation, therapeutic targets

## Abstract

Acute respiratory distress syndrome (ARDS) is characterized by acute diffuse lung injury, which results in increased pulmonary vascular permeability and loss of aerated lung tissue. This causes bilateral opacity consistent with pulmonary edema, hypoxemia, increased venous admixture, and decreased lung compliance such that patients with ARDS need supportive care in the intensive care unit to maintain oxygenation and prevent adverse outcomes. Recently, advances in understanding the underlying pathophysiology of ARDS led to new approaches in managing these patients. In this review, we want to focus on recent scientific evidence in the field of ARDS research and discuss promising new developments in the treatment of this disease.

## Introduction

Normal lung function requires dry alveoli situated closely to perfused capillaries to perform sufficient gas exchange. The capillary endothelium controls the balance between fluids by its selective permeable membrane. This allows serum proteins to stay intravascular and to hold back fluids by oncotic forces. Only small amounts of fluid make it into the interstitium, where it gets reabsorbed by the oncotic gradient or the interstitial lymphatic system.

In acute respiratory distress syndrome (ARDS), acute diffuse lung injury leads to the release of pro-inflammatory cytokines such as tumor necrosis factor alpha (TNFα) and interleukin 1 beta (IL-1β), IL-6, and IL-8, which in turn recruit components of the innate immune system ^1– 4^. Activated neutrophils produce toxic mediators such as reactive oxygen species (ROS) and proteases damaging the endothelium and alveolar epithelium ^5^. Proteins can effuse from the vascular space, which results in the loss of the oncotic gradient between capillaries and the air space, facilitating the advance of fluid into the interstitium and air space ^6^. This protein-rich fluid inactivates surfactant, resulting in the collapse of alveoli. This leads to impaired gas exchange, increased dead space, reduced lung compliance, and decreased carbon dioxide (CO _2_) elimination ^7^.

These acute physiological changes force the patient into a life-threatening situation, making it impossible to maintain the oxygenation and decarboxylation needed. These patients need immediate supportive care in the intensive care unit (ICU). Invasive mechanical ventilation (IMV) is applied in most patients ^8^ with ARDS to guarantee sufficient ventilation and tissue oxygenation, especially for the brain, heart, and kidneys. Although IMV can improve the patient’s condition, IMV itself can damage the vulnerable lung. This ventilator-induced lung injury (VILI) has been known since the beginning of mechanical ventilation in the 1960s and has three major aspects of injury: (1) volutrauma/barotrauma, (2) atelectrauma, and (3) biotrauma.

Volutrauma describes alveolar membrane damage due to over-distention, especially with high tidal volumes rather than high inspiratory pressure (barotrauma), leading to the rupture of the alveoli along with lung edema, cell death, macroscopic pneumothorax, pneumomediastinum, and subcutaneous emphysema ^9^. Atelectrauma, on the other hand, occurs when ventilation with low tidal volumes results in cyclic recruitment and de-recruitment of lung segments. Studies showed that total end-expiratory collapse and reopening of distal airways are accompanied by significant shear stress ^10^ and regional hypoxia followed by cell damage, surfactant dysfunction, inflammation, and pathological progression of the already-injured lung. These mechanical factors of damage are accompanied by the consecutive release of pro-inflammatory mediators, ROS generation ^11, 12^, neutrophil infiltration, immune cell activation ^13^, and, finally, owing to the impaired alveolar–capillary barrier, a translocation of bacteria, toxins, inflammatory cytokines, and activated inflammatory cells into the systemic circulation. This can cause end-organ dysfunction and even death by multiple organ system failure ^14– 17^. This concept is termed biotrauma and may be the primary cause of death in patients with ARDS, as respiratory failure is rarely lethal and most of the time can be sufficiently treated with IMV and extracorporeal decarboxylation and oxygenation. Thus, the management of these patients often represents a tradeoff between maintaining oxygenation and preventing excessive tissue damage.

## Recent advances in ventilation

Patients with ARDS rarely die from respiratory failure ^18^ but rather from the underlying cause during the early period and from pneumonia or sepsis with consecutive multiple organ system failure in the late period ^19^. Although global mortality is still high (40%), survival has improved over the years ^20^. The reasons for the decreasing mortality remain uncertain. Better supportive care, improved ventilation strategies and protocols such as low tidal volume ventilation (LTVV), and the inclusion of trauma-induced ARDS (patients with trauma-induced ARDS have significantly better outcomes than patients with sepsis-induced ARDS) could contribute to the effect observed ^21^.

In 1998, Amato *et al*. published a single-center randomized controlled trial (RCT) (including 53 patients) showing that ventilating ARDS patients with a tidal volume (V _t_) of 6 mL/kg predicted body weight (PBW), a positive end-expiratory pressure (PEEP) above the lowest infliction point on a static pressure–volume curve, and an inspiratory pressure of less than 20 cm H _2_O above PEEP resulted in a significant reduction of 28-day mortality ^22^. These data were validated in 2000 by the Acute Respiratory Distress Syndrome Network (ARMA) trial, which included 861 patients in a multicenter RCT. In this trial, conventional ventilation (V _t_ of 12 mL/kg PBW and plateau pressure of less than 50 cm H _2_O) was compared with protective ventilation (V _t_ of 6 mL/kg PBW and plateau pressure of less than 30 cm H _2_O) ^23^. Protective ventilation resulted in significant reductions in the duration of mechanical ventilation and in-hospital mortality. The oxygen partial pressure/fraction of inspired oxygen (paO _2_/FiO _2_) ratio was lower in the LTVV group compared with conventional ventilation, showing that oxygenation may be a bad surrogate for outcome in ARDS. LTVV improves survival and other clinical outcome parameters by reducing alveolar over-distention ^24, 25^. This beneficial effect is corroborated by a recent meta-analysis of four RCTs ^24^ and a subanalysis of the LUNG SAFE (Large Observational Study to Understand the Global Impact of Severe Acute Respiratory Failure) trial in patients with moderate to severe ARDS showing that peak inspiratory pressure, besides other factors, is a modifiable risk factor for worse outcome ^26^. Interestingly, a prospective cohort study demonstrated that even by raising the V _t_ from 6 to 7 mL/kg PBW, ICU mortality increased by as much as 23% ^27^. Despite this breakthrough, a prospective cohort study showed that LTVV is under-utilized in clinical routine ^28^. This was demonstrated by Bellani *et al*. in 2016 in a multicenter observational study (of 3,022 patients with ARDS) showing a mean V _t_ of 7.5 mL/kg PBW in patients fulfilling ARDS criteria ^8^. Also, only PEEP settings were significantly adapted after ARDS recognition, whereas V _t_ was not ^8^.

It has been discussed that the beneficial effect of protective ventilation may be due to auto-PEEP rather than LTVV ^29^. To maintain minute ventilation in LVVT, the respiratory rate needs to be adapted. Increasing the respiratory rate gives the patient less time to complete expiration, creating auto-PEEP by “air trapping” ^30^. A subanalysis showed that the quantity of auto-PEEP in LTVV is negligible ^31^. However, there are not enough data to judge the role of auto-PEEP on outcome in protective lung ventilation. The ARMA trial investigated whether sedation should be increased to prevent forced breathing work and patient–ventilator asynchrony by using LTVV ^32^. A *post hoc* analysis showed no significant difference in days of sedation or need of opioids or neuromuscular-blocking agents ^33^. However, the ACURASYS (ARDS et Curarisation Systematique) trial showed a beneficial effect on 90-day mortality, incidence of barotrauma, and ventilator-free days when neuromuscular relaxation with cisatracurium was applied in the first 48 hours after onset of ARDS ^34, 35^. The discussed benefits of neuromuscular blockade in ARDS include avoidance of patient–ventilator asynchrony, which lowers transpulmonary pressure and the resulting stress on the lung. It further leads to reduced muscle work with decreased pulmonary oxygen consumption, allowing further reduction of ventilation targets, thereby mitigating VILI and biotrauma. These studies showed that early neuromuscular blockade was not accompanied by increased ICU-acquired weakness. Cisatracurium has anti-inflammatory properties ^36, 37^, though fewer than those of potent anti-inflammatory drugs, which per se did not sufficiently influence outcome in ARDS. Though relatively expensive, cisatracurium seems to be the paralyzing drug of choice in early ARDS, especially because of its favorable pharmacokinetics. Futhermore, cisatracurium is metabolized independently of renal or liver function.

## Driving pressure

Although protective ventilation strategies all aim to reduce stress on the lungs and to avoid volutrauma and barotrauma (for example, LTVV, increasing PEEP, or limiting plateau pressure), changes in one parameter may negatively influence others; for example, increasing PEEP will decrease V _t_ when plateau pressure is limited. Furthermore, V _t_ titrated to PBW may still lead to regional barotrauma, as functional lung size is drastically reduced in patients with ARDS (‘baby lung’ concept) with heterogeneous distribution of aerated and consolidated lung segments. This is reflected in a lowered compliance of the respiratory system (C _RS_).

The normalized V _t_ to actual C _RS_ is termed driving pressure (ΔP = V _t_/C _RS_) and is calculated as the difference between end-inspiratory plateau pressure and PEEP in mechanically ventilated patients. ΔP was significantly associated with increased survival in a pooled retrospective analysis of RCTs focusing on optimal PEEP and V _t_ settings in ARDS. Interestingly, the beneficial effects of restrictive V _t_ or increased PEEP were present only when accompanied by a reduced ΔP ^38^. Whether ΔP is a better predictor for mortality than plateau pressure ^39^ or V _t_ during mechanical ventilation in ARDS needs further clarification.

## Open lung ventilation

Open lung ventilation combines LTVV with optimal PEEP. This results in a decreased over-distension of the alveoli and a reduction of cyclic atelectrauma. Gattinoni *et al*. showed that, even at PEEP levels as high as 45 mm Hg, the percentage of potentially recruitable lung is extremely variable ^40^. Finding the optimal PEEP level for each individual patient may be challenging.

Three major trials compared high versus low PEEP in combination with LTVV with respect to outcome. In the Assessment of Low Tidal Volume and Elevated End-Expiratory Pressure to Obviate Lung Injury (ALVEOLI) trial in 2004, no difference between high and low PEEP was observed ^41^. The Lung Open Ventilation Study (LOVS) trial in 2008 showed improved oxygenation and less need of rescue strategies in the high PEEP group, but no difference in mortality was found ^42^. The Expiratory Pressure (EXPRESS) trial demonstrated more ventilator-free days and more organ failure-free days in the high PEEP group but no difference in mortality ^43^. Currently, there is no universally accepted protocol for open lung ventilation. A meta-analysis of the ALVEOLI, LOVS, and EXPRESS trials in 2010 showed a beneficial effect of high PEEP versus low PEEP only in patients with moderate to severe ARDS ^44^.

In a *post hoc* analysis of the LOVS and EXPRESS trials ^42, 43^, Goligher *et al*. demonstrated that mortality was reduced in patients who were PEEP-responsive compared with patients who were not ^45^. Oxygenation change due to increased PEEP was associated with reduced hospital mortality. The relationship of PaO _2_ to FiO _2_ is also related to cardiac output, oxygen consumption, extrapulmonary shunt, hypoxic pulmonary vasoconstriction, and fractional inspiratory oxygen. The influence of a number of potentially confounding variables on PaO _2_/FiO _2_ may explain the difficulty of LOVS and EXPRESS to demonstrate a significant effect of PEEP on mortality. The appropriate selection of patients (responders and non-responders to PEEP) for RCTs testing PEEP in patients with ARDS may bring new insights.

A trending method to find the optimal PEEP is to calculate the transpulmonary pressure (P _tp_ = alveolar pressure – pleural pressure) by using an esophageal balloon catheter to measure esophageal pressure as a surrogate for pleural pressure. As the pressure to open the lung must overcome chest, abdominal, and alveolar pressure, P _tp_ is representing the force affecting lung distention and stretch. Titrating the PEEP to P _tp_ and maintaining end-inspiratory P _tp_ of less than 25 cm H _2_O reduce cyclic atelectasis and over-distension ^46^. This approach was evaluated by comparing P _tp_-guided PEEP with ARMA trial protocol-guided PEEP. The goal of the study was to maintain the PaO _2_ at 55–120 mm Hg or peripheral capillary oxygen saturation (SpO _2_) at 88–98%. P _tp_-guided PEEP titration resulted in higher PEEP levels and improved PaO _2_/FiO _2_ ratios after 72 hours. No differences in ventilator-free days, ICU stay, or 28-day mortality were found ^46^.

## Cyclic atelectrauma

Rapid changes of oxygen partial pressure caused by cyclic recruitment and derecruitment of atelectasis (CA) during IMV may contribute to lung damage ^47^. Within-breath CA results in varying shunt fractions and altered gas exchange, thereby causing rapid respiratory-dependent alveolar PaO _2_ changes ^48^. These partial pressure of oxygen (PO _2_) oscillations likely cause intermittent hypoxia/hyperoxia injury to the lungs and therefore could represent an additional mechanism of lung and remote organ biotrauma ^49^. Numerous studies have provided evidence for the existence of rapid PaO _2_ changes during respiratory failure ^50– 52^. The changes originate in the diseased lungs and are forwarded with the circulation to the arterial system ^48, 53, 54^. This might promote injury in end organs such as the brain ^55^. Further investigations are needed to validate this separate mechanism of biotrauma in ARDS.

## Prone position

Prone position can be considered to improve oxygenation in patients with ARDS. It optimizes the blood flow to the dependent lung, reduces atelectasis, facilitates secretion drainage, increases functional residual capacity, and reduces plateau pressure. Gattinoni *et al*. showed that patients with ARDS had improved oxygenation when in the prone position ^56^. In this RCT, response to prone position led to a decreased need for PEEP and FiO _2_. Recently, the Proning Severe ARDS Patients (PROSEVA) trial showed a mortality benefit in patients with severe ARDS (16% versus 32.8%) ^57^. In 2010, a further meta-analysis of 10 RCTs demonstrated that there was a significant reduction in mortality in patients with severe ARDS ^58^. However, some trials did not see a beneficial effect of prone position on mortality, especially in patients with moderate or mild ARDS ^59^. PROSEVA excluded patients with mild or moderate ARDS. This might be the reason why PROSEVA could show a survival benefit whereas others could not. Despite some limitations (2,015 patients were not screened, differences in baseline characteristics, and excluding patients if PaO _2_/FiO _2_ was reduced by 20% while in prone position, and so on), the trial indicates the potential benefits of early long proning of patients with severe ARDS in experienced centers.

## Lung imaging

For decades, chest radiographs and computed tomography (CT) have been essential tools in diagnosing and monitoring ARDS. These solid techniques were complemented in recent years with new approaches of functional lung imaging, such as positron emission tomography (PET), lung ultrasound, and electrical impedance tomography (EIT), especially for ventilator adaptation and recruitment during IMV or research purposes ^60, 61^.

At the bedside, lung ultrasound can be used to non-invasively assess pleural effusion, alveolar interstitial syndrome, pneumothorax, lung consolidation, and phenotyping of ARDS and may help to rule out cardiogenic pulmonary edema in the early stages ^62^. It is a cost-effective and easy-to-repeat examination and is more accurate than supine chest radiographs. Cianchi *et al*. ^63^ showed in a small sample of patients with ARDS (12) that the use of lung ultrasound reduced the number of chest radiographs and CT scans, thereby lowering radiation exposure as well as disconnection of ventilators and the potential of adverse events when leaving the ICU ^64, 65^.

PET is helpful in understanding pathophysiologic processes of different phenotypes of ARDS and has been used in numerous clinical ^66^ and experimental ^67^ studies of VILI. Inflammatory and metabolic distribution and activity as well as regional perfusion and function of the lung can be examined by the application of radioactively labeled molecules such as 2-[18F]-fluoro-2-deoxy-D-glucose ([ ^18^F]FDG) or [ ^13^N]nitrogen ( ^13^N _2_). Still, an implementation of PET as a routinely used diagnostic addition to CT scans is limited by availability, especially for modern PET/CT or PET/magnetic resonance imaging scanners.

EIT is a functional imaging technique with great potential in mechanically ventilated patients ^68^. This radiation-free bedside tool measures changes in impedance from differing regional density. It can be used for regional ventilation analysis with dynamic changes in real time.

## Subphenotypes in acute respiratory distress syndrome

A new approach to ARDS defines subphenotypes within ARDS and puts less emphasis on PaO _2_/FiO _2_ ratio and the Berlin definition of ARDS. The heterogeneity of ARDS may contribute to the poor track record of phase 2 and 3 trials of novel therapeutics. It seems that some subgroups of patients benefit more from specific therapeutic approaches than others (for example, early relaxation and low PEEP versus high PEEP). Calfee *et al*. identified subphenotypes within ARDS by applying latent class modeling on two National Heart, Lung, and Blood Institute ARDS RCTs (ARMA and ALVEOLI) ^69^. Phenotype 2 is characterized by higher plasma levels of inflammatory biomarkers, a higher prevalence of vasopressor use, lower serum bicarbonate levels, and higher prevalence of sepsis. Patients with phenotype 2 ARDS had increased mortality and fewer ventilator-free and organ failure-free days. Response to treatment in a randomized trial of PEEP strategy differed on the basis of the subphenotype. Applying high PEEP to phenotype 2 patients resulted in a reduction in mortality. The identification of ARDS subphenotypes may be useful to select patients for specific therapies and assign them to future RCTs ^69^.

## New therapeutic targets

Currently, there are no specific pharmacotherapies evaluated and recommended routinely. Treatment modalities involve supportive care combined with protective ventilation strategies.

## Aspirin

Multiple studies investigated strategies to prevent ARDS in high-risk patients after stratification by the Lung Injury Prediction Score (LIPS). In murine models of acid aspiration-induced lung injury, aspirin could increase oxygenation and decrease neutrophil recruitment and edema. These findings were translated into the LIPS-A trial, a multicenter, double-blinded, placebo-controlled clinical trial. After random assignment, patients with a LIPS of more than 4 received either 325 mg loading dose and 81 mg/day aspirin or placebo for 7 days. The primary endpoint of this trial was the development of ARDS by day 7. The secondary endpoints were ventilator-free days, hospital and ICU length of stay, and 28-day and 1-year survival. There was no significant decrease in the incidence of ARDS at day 7 or in any of the secondary endpoints. This phase 2b trial did not support the continuation to a longer phase 3 trial ^70^.

## β2 agonists

Similar to LIPS-A, the LIPS-B trial investigated whether aerosolized β2-agonists can prevent the development of ARDS. In this multicenter, randomized, double-blinded, placebo-controlled trial, patients with a LIPS of more than 4 received budenosid and formoterol or placebo for 5 days. The primary endpoint was the change in SpO _2_-to-FiO _2_ ratio ^71^. LIPS-B could demonstrate the feasibility of inhaled budenosid and formoterol in patients at high risk for ARDS. Patients who received treatment had improved oxygenation, lower rates of ARDS, and shorter hospital length of stay. LIPS-B is the first prevention trial to suggest efficiency of the intervention for preventing ARDS.

## Stem cells

A further approach to enhance lung tissue repair is the use of stem cells. Animal models showed that stem cells secrete growth factors and cytokines modulating inflammation, promoting tissue repair, and increasing bacterial clearance ^72^. They also may differentiate into mature cells to replace injured tissue. A phase 1 RCT including 12 patients who received allogenic adipose-derived stem cells ^73^ showed an amelioration of epithelial cell injury. The authors claimed that the clinical effect with the doses used was weak and that further optimization is needed. The treatment arm showed a trend toward reduced IL-6 levels at day 5. However, these results were not statistically significant ( *p* = 0.06).

## AP301

The nano-peptide AP301 is a promising new agent in treating ARDS. The structure of the synthetic peptide is based on the lectin-like domain of human TNFα ^74^. AP301 activates the pulmonary epithelial and endothelial sodium channel ENaC that promotes the alveolar liquid clearance ^75^. The beneficial effect of AP301 was demonstrated in animal models of pulmonary edema ^76^. Recently, a phase 1 single-center randomized clinical trial including 48 male volunteers was assessed ^77^. Oral inhaled AP301 was well tolerated by all participants and no adverse effects were reported ^77^. Krenn *et al*. performed a double-blind, placebo-controlled trial including 40 patients with ARDS treated with inhaled AP301 or placebo for 7 days ^78^. There was no difference in mean baseline-adjusted extravascular lung water index between the groups. An exploratory *post hoc* subgroup analysis indicated reduced extravascular lung water index in patients with Sequential Organ Failure Assessment (SOFA) scores of at least 11 receiving AP301 ^78^.

## SB-681323

SB-681323 is a further agent directly targeting the pathophysiological process underlying ARDS. This selective p38 alpha inhibitor may dampen the inflammatory response by interfering with the mitogen-activated protein kinase (MAPK) pathway. This pathway is subsequently involved in the activation of cytokines. A phase 2a multicenter study is investigating the safety and tolerability of intravenous SB-681323 in patients at risk of developing ARDS and evaluates its efficiency by measuring biomarkers linked to the p38 alpha regulatory mechanisms ^79^.

## Interferon beta-1a

Interferon beta-1a induces the upregulation of cluster of differentiation 73 (CD 73). This molecule is responsible for the endothelial barrier function and leads to the prevention of vascular leakage, the main pathophysiological event in ARDS. Treatment with interferon beta-1a was associated with an 81% reduction in odds of 28-day mortality ^80^. However, these findings need to be validated in larger prospective RCTs. Currently, a phase 3 clinical trial comparing the efficiency of interferon beta-1a compared with placebo control is underway.

## MicroRNA

MicroRNAs (miRNAs) are small non-coding RNAs involved in the post-transcriptional regulation of various genes’ expression ^81^. Recently, Cardinal-Fernández *et al*. suggested a few miRNAs as disease biomarkers and therapeutic agents in ARDS ^82^. In 2012, Cai *et al*. investigated the effect of miRNA in a lipopolysaccharide (LPS)-induced acute lung injury mouse model ^83^. The miRNA miR-16 was significantly downregulated in LPS-induced lung injury. Cell culture experiments demonstrated that miR-16 binds to the 3'-untranslated region (3'-UTR) of IL-6 and TNFα and significantly downregulates their expression levels ^84^. Further investigations revealed that miR-16 was downregulated in mice exposed to hyperoxia ^85^. The authors exposed A549 cells to hyperoxia and overexpressed miR-16. This resulted in increased ENaCβ levels and the suppression of transforming growth factor-beta (TGF-β), an inhibitor of ENaC. This study suggests that miR-16 promotes the resolution of pulmonary edema in ARDS by enhancing the expression of ENaC.

In a further study, miR-127 was downregulated in LPS-induced lung injury. An *in vivo* lung injury model showed that overexpression of miR-127 leads to reduced pulmonary vascular permeability, inflammatory cell infiltration, cytokine levels, and activation of complement and STAT3 (signal transducer and activator of transcription 3) signaling ^86^.

As mentioned above, granulocyte-macrophage colony-stimulating factor (GM-CSF) enhances the repair mechanisms in injured lung tissue and increases the alveolar macrophage function ^87^. In response to hyperoxia, epithelial cells express high miR-133 levels ^88^, leading to the suppression of GM-CSF. Manipulations on miR-133 may provide novel interventions to reduce lung injury.

In addition to pulmonary endothelial and epithelial tissue damage, macrophages play an important role in promoting and resolving inflammation ^89^. Alternatively, activated macrophages can participate in the resolution of inflammation in ARDS by GM-CSF ^89^. miRNAs may control signaling pathways contributing to macrophage phenotypes. miR-155, miR-127, and miR-429 regulate their targets to promote a pro-inflammatory macrophage phenotype ^90, 91^. Let-7e, miR-127, miR-320b, and miR-146a promote an immunosuppressor macrophage phenotype ^86, 92– 94^. Interestingly, miR-429 promotes the pro-inflammatory macrophage phenotype by the inhibition of the dual specificity protein phosphatase 1 (DUSP-1) protein. Like SB-681323 mentioned earlier, DUSP-1 protein is a key player in p38 MAPK signaling pathways.

Furthermore, circulating miRNAs could represent novel biomarkers for ARDS. Sun *et al*. demonstrated that circulating miR-181b levels were reduced in ARDS patients compared with controls ^95^. The authors also showed that the substitution of miR-181b reduced lung injury and mortality in a mouse lung injury model ^95^. A further study concluded that miR-125b may be a promising biomarker in patients with ARDS. Increased miR-125b levels resulted in an improvement of lung function in LPS-injured mice ^96^. Despite these findings, the prognostic value of circulating miRNAs in ARDS remains unexplored.

## C5a

C5a is a complement cleavage product acting as a potent anaphylatoxin promoting the appearance of neutrophil extracellular traps (NETs). NETs consist of nuclear chromatin, containing histones and other nuclear proteins. These extracellular histones may trigger the release of TGF-β via granules of platelets and therefore be involved in tissue remodeling during ARDS. NETs mediate apoptosis in epithelial and endothelial cells and the production of inflammatory mediators during ARDS ^97^. Interception of NETs showed promising beneficial effects in different lung injury models. Administering neutralizing antibodies directed against NETs reduced pulmonary vascular permeability and the volume of extravascular lung water ^98^. In a murine lung injury model, the degradation of NET structures with DNAse1 reduced lung injury and mortality ^98^. Eculizumab is a monoclonal anti-C5 antibody approved for the treatment of hemolytic uremic syndrome and paroxysmal nocturnal hemoglobinuria ^99^. Eculizumab may block C5-induced NET release in ARDS, leading to improved outcomes.

## Conclusions

Recent advances in understanding the pathophysiology of ARDS have led to improved outcomes. LTVV combined with open lung strategies are the hallmarks of ARDS management and have contributed to new insights in understanding lung injury. Prone position and neuromuscular relaxation may be beneficial in selected patients. Currently, no pharmacological treatments exist, although promising ongoing trials are being assessed. Studying the regulatory roles of miRNAs may contribute to a better understanding of how to control inflammatory target gene expression in ARDS. Modulation of miRNA expression could represent a novel therapeutic approach to treat the underlying cause of ARDS.

## Abbreviations

ΔP, driving pressure; ARDS, acute respiratory distress syndrome; CA, cyclic atelectasis; C _RS_, respiratory system compliance; CT, computed tomography; DUSP-1, dual specificity protein phosphatase 1; EIT, electrical impedance tomography; ENaC, epithelial sodium channel; FiO _2_, fraction of inspired oxygen; GM-CSF, granulocyte-macrophage colony-stimulating factor; ICU, intensive care unit; IL, interleukin; IMV, invasive mechanical ventilation; LIPS, Lung Injury Prediction Score; LPS, lipopolysaccharide; LTVV, low tidal volume ventilation; MAPK, mitogen-activated protein kinase; miRNA, microRNA; NET, neutrophil extracellular trap; PaO _2_, oxygen partial pressure; PBW, predicted body weight; PEEP, positive end-expiratory pressure; PET, positron emission tomography; P _tp_, transpulmonary pressure; RCT, randomized controlled trial; ROS, reactive oxygen species; SpO _2_, peripheral capillary oxygen saturation; TGFβ, transforming growth factor beta; TNFα, tumor necrosis factor alpha; VILI, ventilator-induced lung injury; V _t_, tidal volume; ARMA, Acute Respiratory Distress Syndrome Network trial; ALVEOLI, Assessment of Low Tidal Volume and Elevated End-Expiratory Pressure to Obviate Lung Injury trial; LOVS, Lung Open Ventilation Study; EXPRESS, Expiratory Pressure Study; PROSEVA, Proning Severe ARDS Patients trial.
